# Phenotypic dissection of the mouse *Ren1d* knockout by complementation with human renin

**DOI:** 10.1074/jbc.RA117.000160

**Published:** 2017-11-09

**Authors:** Charlotte Buckley, Robert J. Nelson, Linda J. Mullins, Matthew G. F. Sharp, Stewart Fleming, Christopher J. Kenyon, Sabrina Semprini, Dominik Steppan, Janos Peti-Peterdi, Armin Kurtz, Helen Christian, John J. Mullins

**Affiliations:** From the ‡ University/BHF Centre for Cardiovascular Science, University of Edinburgh, Edinburgh EH16 4TJ, United Kingdom,; the ¶ University of Dundee, Ninewells Hospital Medical School, Dundee DD1 9SY, Scotland,; the ‖ Physiologisches Institut der Universität Regensburg, Regensburg D-93053, Germany,; the ** Department of Physiology and Biophysics and Zilkha Neurogenetic Institute, Keck School of Medicine, University of Southern California, Los Angeles, California 90089, and; the ‡‡ Department of Physiology, Anatomy and Genetics, University of Oxford, Oxford OX1 3QX, United Kingdom

**Keywords:** granulation, human renin, juxtaglomerular, macula densa, renin, renin angiotensin system, electron microscopy (EM), confocal microscopy, immunochemistry, mouse, secretion, animal model, transgenic mice

## Abstract

Normal renin synthesis and secretion is important for the maintenance of juxtaglomerular apparatus architecture. Mice lacking a functional *Ren1d* gene are devoid of renal juxtaglomerular cell granules and exhibit an altered macula densa morphology. Due to the species-specificity of renin activity, transgenic mice are ideal models for experimentally investigating and manipulating expression patterns of the human renin gene in a native cellular environment without confounding renin–angiotensin system interactions. A 55-kb transgene encompassing the human renin locus was crossed onto the mouse *Ren1d*-null background, restoring granulation in juxtaglomerular cells. Correct processing of human renin in dense core granules was confirmed by immunogold labeling. After stimulation of the renin–angiotensin system, juxtaglomerular cells contained rhomboid protogranules with paracrystalline contents, dilated rough endoplasmic reticulum, and electron-lucent granular structures. However, complementation of *Ren1d*^−/−^ mice with human renin was unable to rescue the abnormality seen in macula densa structure. The juxtaglomerular apparatus was still able to respond to tubuloglomerular feedback in isolated perfused juxtaglomerular apparatus preparations, although minor differences in glomerular tuft contractility and macula densa cell calcium handling were observed. This study reveals that the human renin protein is able to complement the mouse *Ren1d*^−/−^ non-granulated defect and suggests that granulopoiesis requires a structural motif that is conserved between the mouse *Ren1d* and human renin proteins. It also suggests that the altered macula densa phenotype is related to the activity of the renin-1d enzyme in a local juxtaglomerular renin–angiotensin system.

## Introduction

The renin–angiotensin system plays a major role in the regulation of blood pressure and electrolyte balance through the production of the vasoactive peptide hormone angiotensin II (Ang-II).^4^ Renin catalyzes the conversion of angiotensinogen to angiotensin I (Ang-I), which is then cleaved by angiotensin-converting enzyme (ACE) to produce Ang-II. Renin, generally considered to be the rate-limiting step in the angiotensin cascade, is predominantly produced in the juxtaglomerular (JG) cells of the kidney. Within these cells, renin is packaged and stored within membrane-bound electron-dense granules, although the factors and dynamic processes governing the onset and maturation of granules remain to be fully elucidated (1). For example, recent stereological reconstructions of serial electron micrographs of JG cells suggest that granules may exist as part of larger vesicular structures of varying complexity, which appear to be dependent on the secretory stimulation of the cell (2, 3).

Human and rat genomes contain a single gene for renin, but mouse strains vary in renin gene copy number (4, 5); some strains, *e.g.* CBA/Ca, possess the single ancestral gene *Ren1c*, whereas others, *e.g.* 129/Sv, possess two renin genes, termed *Ren1d* and *Ren2*. The *Ren1d* and *Ren2* genes share the same chromosomal locus, being located 21 kb apart on mouse chromosome 1 (6, 7), and have the same overall genomic organization (8, 9), sharing 96% sequence identity within the coding region and encoding highly homologous, but distinct, proteins.

Gene knock-out studies on the mouse renin locus found that *Ren2*-null mice had no significant cardiovascular phenotype (10), although animals did have increased concentrations of active renin and decreased prorenin in the plasma. Investigation of *Ren1d* knock-out mice revealed a sexually dimorphic hypotension in female *Ren1d*^−/−^ mice (11). In contrast to *Ren2*^−/−^ mice, *Ren1d*^−/−^ animals had increased plasma concentrations of inactive prorenin and decreased circulating active renin. However, most striking was the complete absence of renin storage/secretory granules in the JG cells of the renal afferent arteriole in *Ren1d*^−/−^ animals. These studies suggested that expression of *Ren2* could partially compensate for the loss of *Ren1d* with respect to maintaining arterial blood pressure but was unable to maintain normal juxtaglomerular apparatus structure.

In transgenesis experiments with a 145-kb BAC construct spanning both the mouse *Ren2* and *Ren1d* loci, homozygous *Ren1d*-null mice expressing the BAC clone exhibited complete restoration of normal renal structure (12). The introduction of a modified 145-kb BAC transgene, in which the *Ren1d* locus was disrupted by introduction of a β-galactosidase-neomycin resistance (β-geo) reporter, onto the *Ren1d* knock-out background demonstrated reporter gene expression in the absence of renin-1d. Taken together, these experiments confirm that expression of the renin-1d protein is a prerequisite for granule formation and maturation, and that the *Ren2* gene product is unable to act as substitute in this role.

In addition, a discrete and reproducible change was observed in the cell number and morphology of the macula densa (MD) cells of the kidney distal tubular epithelium of *Ren1d*^−/−^ mice (11). Extensive cross-talk between the flow- and salt-sensing MD plaque of the cortical thick ascending limb and JG cells plays a significant role in the secretion of renin as part of tubuloglomerular feedback (TGF). TGF signaling acts to fine-tune single nephron glomerular filtration rate (GFR) in response to fluctuating distal tubular sodium concentrations; low sodium induces renin secretion, whereas high sodium at the MD plaque inhibits renin secretion, causes vasoconstriction of the afferent arteriole (AA), and a decline in single nephron GFR (13). It was not clear whether the altered cellular morphology in the absence of renin-1^d^ was indicative of altered TGF functionality.

Transgenic mice are ideal models for experimentally investigating and manipulating expression patterns of the human renin gene in a native cellular environment without the need for human tissue (13). Renin activity is species-specific (14), allowing independent determination of plasma and tissue levels of mouse and human renin, and preventing interaction of human renin with the murine RAS (13).

We report the dissection of the *Ren1d* knock-out mouse phenotypes by complementation with the human renin gene. Granulation of JG cells was restored in a transgene expression level-dependent manner, with higher levels of renin expression correlating with increased granular number and volume. Complementation did not restore MD morphology, which retained the same hypercellular and columnar appearance as *Ren1d*^−/−^ mice. However, this altered morphology was not indicative of MD function; both *Ren1d*^−/−^ mice and human renin complemented mice were able to perform TGF effectively in isolated perfused juxtaglomerula apparatus (JGA) preparations.

## Results

### Derivation of human renin transgenic lines

**Figure 1.**
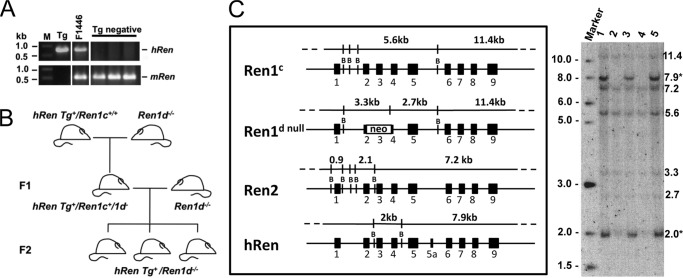
*A,* transgene positive animals were identified by PCR. Marker (*M*), New England Biolabs 1-kb DNA Ladder; *Tg*, PAC 111L11; *F1446*, *hRen*^+^; *Tg negative*: transgenic negative animals. *B,* breeding strategy to move the *hRen* transgene from a *Ren1c* background to the *Ren1d* ^−/−^ background. *C,* the structure of the mouse and human renin genes and the predicted sizes of BamHI fragments that are detected by a mouse *Ren2* cDNA hybridization probe are shown in the *left panel*. The autoradiograph (*right*) is an exemplary Southern blot showing the genotyping of F1 backcross progeny. Inheritance of the human renin transgene is indicated by the presence of diagnostic human-specific fragments of 2.0 and 7.9 kb (*asterisks*).

A 55-kb fragment of PAC 111L11, transgene *hRen*, containing ∼35 kb of 5′-flanking and 10 kb of 3′-flanking sequences was used to generate transgenic mice by pronuclear microinjection. Two of four founder mice were backcrossed to *Ren1d*^−/−^ mice (Fig. 1, *A* and *B*) and *hRen*^+/−^*Ren1d*^−/−^ mice were used for detailed pheotypic analyses. Progeny from both founders showed similar results and line 1446 was expanded and used for more detailed study. Diagnostic Southern blot hybridization was used to confirm inheritance of the human renin transgene by the presence of 2.0- and 7.9-kb human-specific fragments (Fig. 1*C*).

### Complementation of the Ren1d knock-out with human renin

**Figure 2.**
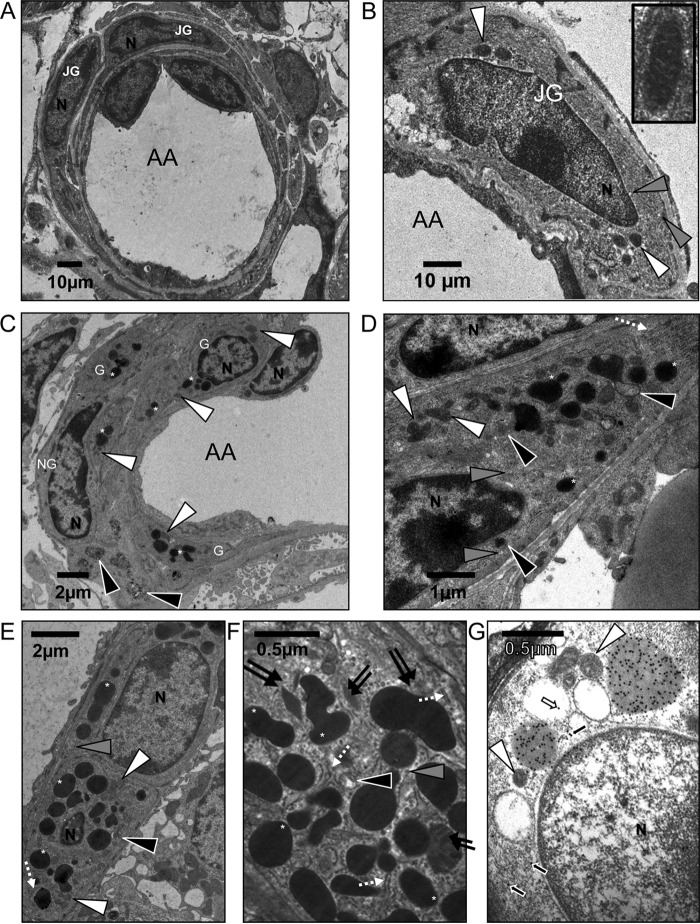
**Ultrastructure of kidney juxtaglomerular cells from *hRen*^+/−^*Ren1d*^−/−^ and *Ren1d*^−/−^ mice.** For all images: *G,* granulated JG cell; *NG,* non-granulated JG cell; *N,* nucleus; *white arrowhead,* mitochondria; *gray arrowhead*, electron-lucent vesicle; *black arrowhead*, immature renin-containing granules; *dashed white arrow,* rough endoplasmic reticulum; *double black arrow*, paracrystalline protogranule; *white asterisk*, dense core renin-containing granule. *A,* low magnification image of afferent arteriole surrounded by juxtaglomerular cells in a *Ren1d*^−/−^ mouse, showing no electron dense granules. *B,* high magnification juxtaglomerular cells from *Ren1d*^−/−^ mice, containing no granules. *Inset* shows high magnification image of mitochondria. *C,* low magnification, sparse granulation of male *hRen*^+/−^*Ren1d*^−/−^ juxtaglomerular cells surrounding the afferent arteriole. *G,* granulated JG cells; *NG,* non-granulated JG cell. *D,* high magnification ultrastructure of male *hRen*^+/−^*Ren1d*^−/−^ juxtaglomerular cells showing sparse granulation. *E* and *F,* highly granulated female *hRen*^+/−^*Ren1d*^−/−^ juxtaglomerular cell. *G,* anti-*hRen* immunogold-labeled sections from male *hRen*^+/−^*Ren1d*^−/−^ juxtaglomerular cells. *White arrows*, immunogold labeling within electron-lucent granules; *black arrows*, immunogold labeling within cytoplasm. *Scale bars* are represented individually.

The granulation status of renal JG cells was assessed by 2D transmission electron microscopy (TEM). Examination of the subcellular structure of *Ren1d*^−/−^ mice confirmed the absence of electron-dense secretory granules in the JG cells of *Ren1d*^−/−^ mice (Fig. 2, *A* and *B*), as previously described (11). The only visible subcellular organelles were small cytoplasmic electron-lucent vesicles (*gray arrowheads*) and mitochondria (*white arrowheads*). This is illustrated in the magnified inset, where the characteristic lines associated with mitochondria can be seen (Fig. 2*B*).

In contrast, TEM examination of the subcellular structure of *hRen*^+/−^*Ren1d*^−/−^ renal JG cells revealed the presence of dense-core secretory granules in both males (Fig. 2, *C* and *D*) and females (Fig. 2, *E* and *F*). Granule electron density was seen to be uniform, particularly in female *hRen*^+/−^*Ren1d*^−/−^ mice (Fig. 2, *E* and *F*, *white asterisks*). As well as the dense core granules (*white asterisks*) and mitochondria (*white arrowheads*), rough endoplasmic reticulum (*dashed white arrow*), small electron-lucent vesicles (*gray arrowheads*), and immature granules (*black arrowheads*) were visible. Paracrystalline protogranules (*double arrows*) were also occasionally present in the Golgi apparatus (Fig. 2, *E* and *F*). Confirmation of the presence of active human renin within dense core granules of *hRen*^+/−^*Ren1d*^−/−^ JG cells was performed on ultrathin sections using immunogold labeling (Fig. 2*G*). Staining was observed overwhelmingly within the dense core granules (∼88%, *white asterisks*), with no staining within the mitochondria (*white arrowheads*). A small subset of immunogold particles stained the cytoplasm (∼12%, black arrows) and there was only a single instance of staining within electron-lucent granules (*white arrow*).

**Figure 3.**
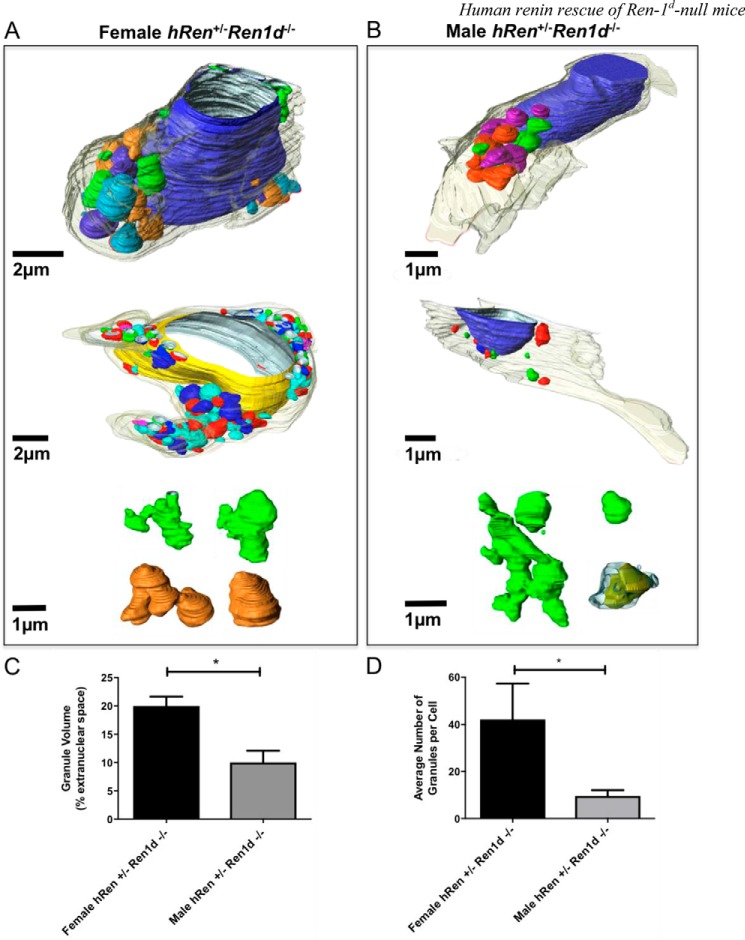
**3D reconstruction of juxtaglomerular cells from *hRen*-complemented *Ren1d*^−/−^ mice.** Reconstructions of serial electron micrographs were rendered using Amira (FEI) software for female (*A*) and male (*B*) *hRen*^+/−^*Ren1d*^−/−^ juxtaglomerular cells. The nucleus is represented in *blue* or *yellow*. Granules are colored to allow ease of differentiation between solitary or networked granules. *C,* the volume of the rendered granules was assessed with respect to the percentage of extranuclear space and compared between female and male *hRen*^+/−^*Ren1d*^−/−^ mice. *D,* the average number of granules was also assessed and compared between male and female *hRen*^+/−^*Ren1d*^−/−^ animals. *Error bars* are mean ± S.E. from cells with *n* > 3 in each group. *, *p* < 0.05; **, *p* < 0.01 by Student's *t* test. *Scale bars* are represented individually.

Many more large dense granules (*white asterisks*) were visible in JG cells from females (Fig. 2, *E* and *F*) when compared with those from male *hRen*^+/−^*Ren1d*^−/−^ mice (Fig. 2, *C* and *D*). To quantitatively compare granulation levels between male and female *hRen*^+/−^*Ren1d*^−/−^ mice, serial sections were imaged using TEM. The granules, membrane, and nucleus were manually segmented within the image to generate faithful 3D reconstructions of JG cells and their intracellular organelles (Fig. 3). Although rare instances of linkages between granules were seen in female *hRen*^+/−^*Ren1d*^−/−^ mice (Fig. 3*A*), the majority were regular in size and shape, existing as solitary granules rather than as part of a larger network. These took up on average 20% extranuclear space compared with the 10% cytoplasmic volume of granules in JG cells from male *hRen*^+/−^*Ren1d*^−/−^ mice (Fig. 3*C*). The granules from this group were irregular in shape and size and significantly less densely packed (Fig. 3*B*). Granules showed non-uniform electron density, and the number of granules per cell was more variable (Fig. 3*B*, *bottom panel*). The average granule number in JG cells from male *hRen*^+/−^*Ren1d*^−/−^ mice was also significantly lower than those found in females (Fig. 3*D*).

### ACE inhibition in human renin-complemented Ren1d-null mice

To determine whether *hRen* transgene expression levels could be regulated by pharmacological manipulation and whether granulation was correlated with the transgene expression level, groups of mice were treated with the ACE inhibitor, captopril. Male and female *hRen*^+/−^*Ren1d*^−/−^ mice were subjected to captopril or vehicle treatment for 10 days. Following this period, plasma was collected to measure plasma active human renin and mouse renin levels. Mice were sacrificed and kidney tissue collected for histological examination and to assess transgene expression by quantitative real-time PCR.

**Figure 4.**
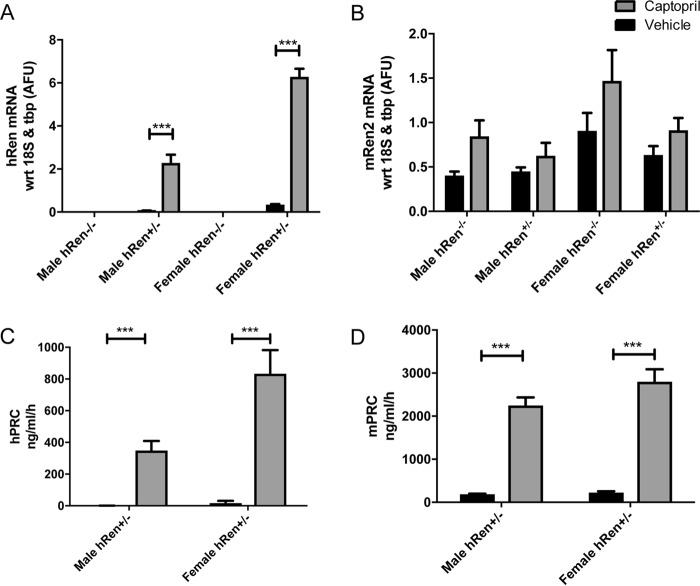
*A* and *B*, whole kidney assessment of human renin (*A*) and mouse renin (*B*) transcript levels in male and female *hRen*^+/−^*Ren1d*^−/−^ and *hRen*^−/−^ *Ren1d*^−/−^ untreated or captopril-treated (1 mg/ml in the drinking water for 10 days, *n* = 6) mice. *C,* human plasma renin concentration (*hPRC*), and *D,* mouse plasma renin concentration (*mPRC*) in response to ACE inhibition with captopril or treatment with vehicle in male and female *hRen*^+/−^*Ren1d*^−/−^ mice (*n* = 3–7). *Error bars* represent S.E. with *, *p* < 0.05; **, *p* < 0.01; ***, *p* < 0.001; ****, *p* < 0.0001 by two-way analysis of variance in conjunction with Bonferroni post hoc analysis.

Quantitative PCR confirmed that human renin was not expressed in *Ren1d* ^−/−^ mice, either at baseline or after treatment with captopril (Fig. 4*A*). In *hRen*^+/−^*Ren1d*^−/−^ mice, expression of the *hRen* transgene was higher in females compared with males at baseline (11-fold). Captopril treatment led to a significant increase in *hRen* expression in males (93-fold) and females (22-fold) (Fig. 4*A*). Expression of *mRen2* tended to show a slight increase after captopril treatment but did not significantly differ between genotypes of each sex (Fig. 4*B*). However, human and mouse plasma renin concentrations increased dramatically after inhibition in both males and females (Fig. 4, *C* and *D*).

### Subcellular structure following ACE inhibition

**Figure 5.**
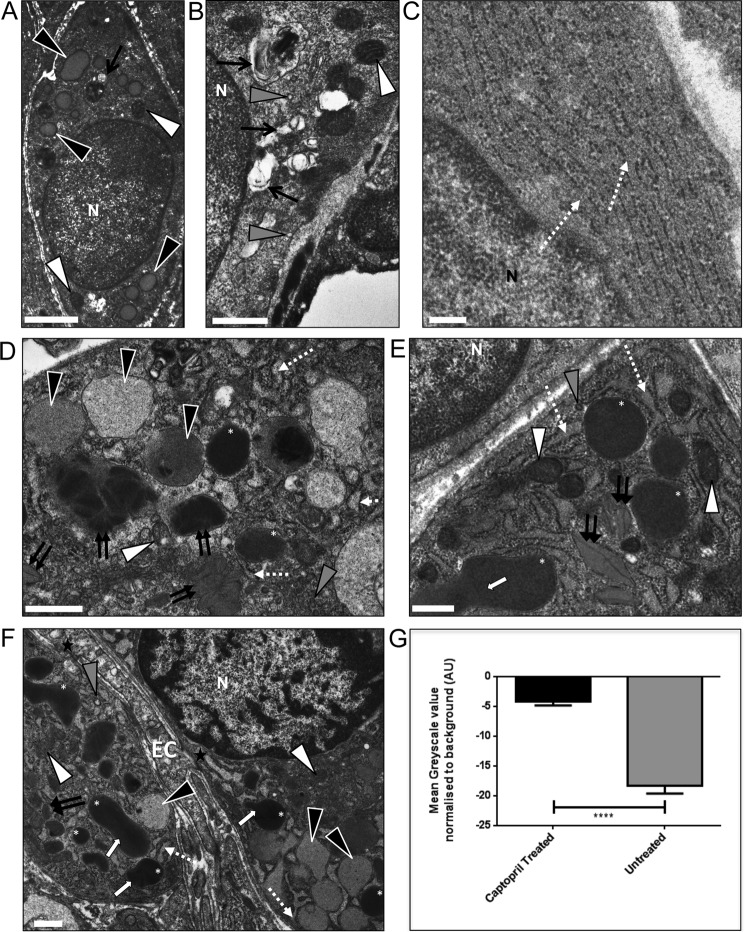
**Ultrastructure of juxtaglomerular cells in *Ren1d*^−/−^ and *hRen*^+/−^*Ren1d*^−/−^ mice after ACE inhibition.** Animals were administered captopril (1 mg/ml in drinking water for 10 days). For all images, *N,* nucleus; *white arrowheads*, mitochondria; *gray arrowheads*, electron-lucent vesicles; *black arrowheads*, electron-lucent granules; *single black arrow*, electron-lucent lysosomes; *white arrows*, granule merging; *dashed white arrow*, rough endoplasmic reticulum; *double black arrow*, protogranules containing paracrystalline material; *EC*, endothelial cells; *black star*, gap junction; *white asterisk*, dense core renin-containing granule. *Scale bars* represent 500 nm. *A–C, Ren1d*^−/−^ mice. *D–F, hRen*^+/−^*Ren1d*^−/−^ male mice. *G,* granule lucency was measured in 8 cells from 8 different mice per group, where the mean pixel intensity was analyzed with respect to the electron density of the cytosol. ****, *p* < 0.0001 by Student's *t* test. *Error bars* represent S.E.

TEM was performed to examine the subcellular structure of the *Ren1d*^−/−^ and *hRen*-complemented *Ren1d*^−/−^ JG cells following ACE inhibition. In *Ren1d*^−/−^ mice, large, electron-lucent granules were visible (Fig. 5*A*, *black arrowheads*), as were mitochondria (Fig. 5, *A* and *B*, *white arrowheads*) and lysosomal-type structures (Fig. 5*B*, *black arrow*). Furthermore, many small vesicles were identifiable in the cytosol (Fig. 5*B*, *gray arrowheads*), with a prominent RER visible after captopril treatment (Fig. 5*C*, *dashed white arrow*).

Higher levels of granulation were evident after ACE inhibition in male *hRen*^+/−^*Ren1d*^−/−^ mice, along with vastly dilated RER (Fig. 5, *D–F*, *dashed white arrow*), suggesting increased renin protein synthesis. Interestingly, captopril treatment induced the formation of electron-lucent (*black arrowheads*) as well as electron-dense (*white asterisks*) granules within *hRen*^+/−^*Ren1d*^−/−^ JG cells (Fig. 5, *D–F*). These granules varied in both electron-lucency and size, from small granules surrounded by dilated RER to larger, merged dense-core granules (Fig. 5, *E* and *F*, *white arrows*). To quantify this, the mean pixel intensity was determined for each granule and normalized to the cytosol mean pixel intensity. Using this method, the darker and more electron-dense the granule the more negative the normalized mean pixel value. Comparing this value in untreated and captopril-treated *hRen*^+/−^*Ren1d*^−/−^ JG cells clearly showed that the granules were, on average, more electron-lucent in JG cells from animals that have undergone ACE inhibition (Fig. 5*G*). Images also strongly suggested that coalescence of paracrystalline material within granules and immature granules were present to a greater degree after captopril treatment than at baseline (Fig. 5, *D–F*, *double black arrows*).

### Macula densa morphology

**Figure 6.**
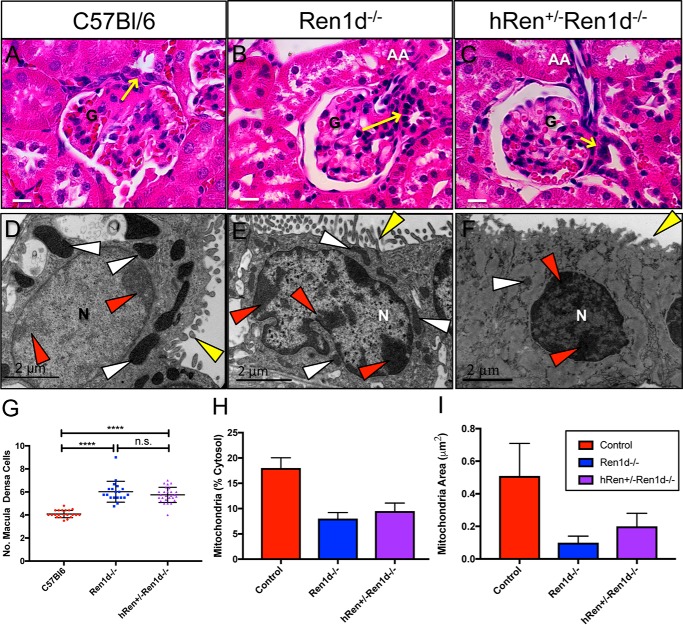
**Macula densa morphology remains altered in *hRen* rescue mice on a *Ren1d*^−/−^ background.** *A*–C, H&E-stained kidneys sections showing the macula densa at its JGA position from male C57Bl/6, *Ren1d*^−/−^, and *hRen*^+/−^*Ren1d*^−/−^ mice. *Yellow arrow*, macula densa plaque; *N,* nucleus; *G,* glomerular tuft. *Scale bars* represent 20 μm. *D–F*, electron micrographs of macula densa cells in C57Bl/6, *Ren1d*^−/−^, and *hRen*^+/−^*Ren1d*^−/−^ mice. *N,* nucleus; *white arrowheads*, mitochondria; *red arrowheads*, dense packed chromatin; *yellow arrowheads*, microvilli. *Scale bars* represent 2 μm. *G,* quantification of macula densa cell number in a plaque (*n* = 4 *Ren1d*^−/−^; *n* = 5 C57Bl/6, *hRen*^+/−^*Ren1d*^−/−^). ****, = *p* < 0.0001. *H* and *I,* quantification of a cytoplasmic area occupied by mitochondria, and mitochondrial area in C57Bl/6, *Ren1d*^−/−^, and *hRen*^+/−^*Ren1d*^−/−^ mice.

Consistent with previous findings (11), H&E histological examination of JGA from *Ren1d*^−/−^ mice demonstrated a hypercellularity of the MD plaque when compared with heterozygous C57Bl/6 mice (Fig. 6, *A–C* and *G*). *hRen*^+/−^*Ren1d*^−/−^ mice retained this MD hypercellularity and altered cell morphology, showing significantly more MD cells in the plaque (*yellow arrows*), compared with C57Bl/6 controls. To further investigate intracellular ultrastructure, TEM images of MD were acquired and structural cellular parameters quantified and compared with C57Bl/6 controls (Fig. 6, *D–E*, *H*, and *I*).

Control C57Bl/6 MD cells were characterized by invaginations of the plasma membrane along the border with JG cells, which was not evident in distal tubule cells on the opposite face of the tubule. In contrast, in *Ren1d*^−/−^ and *hRen*^+/−^*Ren1d*^−/−^ MD cells there was little invagination of the plasma membrane but intercellular spaces between MD cells were large. In *Ren1d*^−/−^ and *hRen*^+/−^*Ren1d*^−/−^ MD cells, mitochondria (*white arrowheads*) were small and sparsely distributed in the cell compared with the large, dilated mitochondria found in C57Bl/6 MD cells (*p* < 0.05). There also appeared to be a difference in MD nuclear chromatin between groups; a greater amount of dense nuclear chromatin (*red arrowheads*) was seen in *Ren1d*^−/−^ groups compared with the pale chromatin indicative of dilated chromatin in C57Bl/6 controls. However, the appearance of microvilli (*yellow arrowheads*) and intercellular junctions between MD cells did not appear to differ between groups.

**Figure 7.**
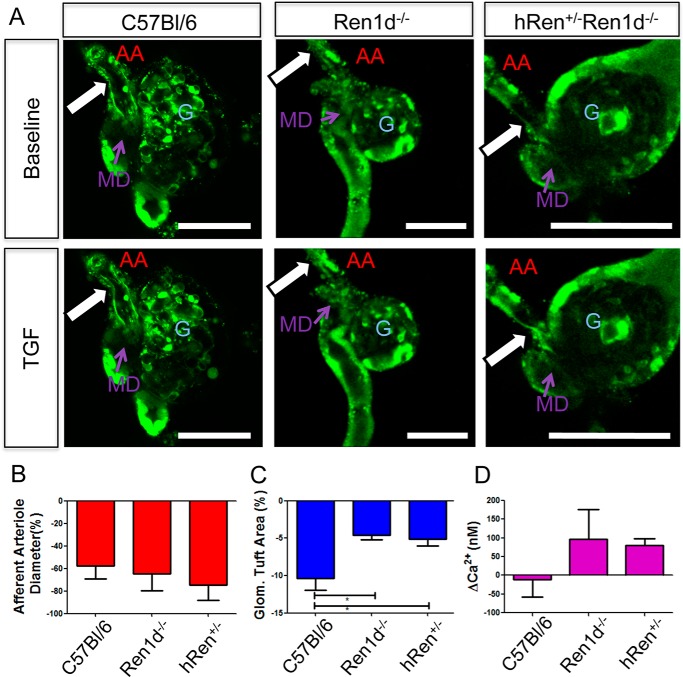
**Tubuloglomerular feedback functions appropriately in isolated perfused juxtaglomerular apparatus from C57Bl/6, *Ren1d*^−/−^, and *hRen*^+/−^*Ren1d*^−/−^ mice.** Flow-induced TGF was triggered by increasing the rate of constant 10 mm NaCl-containing tubular perfusion from 2 to 20 nl/min. *A,* TGF is manifested by constriction of the glomerular tuft area and afferent arteriole diameter in C57Bl/6, *Ren1d*^−/−^, and *hRen*^+/−^*Ren1d*^−/−^ mice. *G,* glomerulus. Glomerular architecture was visualized and assessed using ratiometric calcium indicator dyes Fluo-4 (*green*) and Fura Red (*red*, not shown). *Scale bars* represent 100 μm. *White arrows* indicate the location of AA constriction at baseline and after triggering TGF. TGF response was quantified by measuring the change in (*B*) AA diameter, (*C*) glomerular tuft area, and (*D*) calcium concentration at the macula densa plaque. *Error bars* = S.E., *, *p* < 0.05; **, *p* < 0.01 using Student's *t* test. *n* > 3 in each group.

To determine whether this altered morphology was indicative of impaired TGF in the juxtaglomerular apparatus environment, freshly dissected C57Bl/6, *Ren1d*^−/−^, and *hRen*^+/−^*Ren1d*^−/−^ glomerular tufts with attached afferent arteriole, cTAL and MD plaques were microperfused. Either perfusate flow (Fig. 7) or NaCl concentration (data not shown) was varied at the MD plaque, and the TGF responses were measured via elevations in intracellular [Ca^2+^]*_i_* and mechanical responses of the glomerular tuft and afferent arteriole, as previously described (15).

All groups demonstrated appropriate TGF in response to both stimuli; afferent arteriole contraction (Fig. 7*A*, *white arrows*) and glomerular tuft contraction were seen in response to the triggered TGF, and calcium waves were seen to move throughout the JGA preparation. Quantification of these responses showed no difference in response of the afferent arteriole contraction (Fig. 7*B*), however, the change in glomerular tuft area was slightly blunted in both *Ren1d*^−/−^ or *hRen*^+/−^*Ren1d*^−/−^ groups (Fig. 7*C*).

No significant differences in [Ca^2+^]*_i_* elevations through vascular smooth muscle cell, podocytes, cortical thick ascending limb or JG cells in the JGA were detected (data not shown). In MD cells, more [Ca^2+^]*_i_* propagated through MD cells in both the *Ren1d*^−/−^ and *hRen*^+/−^*Ren1d*^−/−^ groups compared with the C57Bl/6 group (Fig. 7*D*), although this was not significant. This effect was heterogeneous, seen only in certain MD cells in the plaque.

## Discussion

Previous data have revealed that the presence of the renin-1^d^ protein is essential for granule formation in two renin gene mice (11, 12). We now show that complementation of the *Ren1d* knock-out mouse with human renin results in a restoration of the granulation in the JG cells of the kidney in a transgene expression level-dependent manner. These observations are consistent with the hypothesis that the human and mouse renin-1d proteins have conserved structural features necessary for the trafficking of the renin proteins to the regulated pathway of secretion, and thus the formation of dense renin-containing granules. Immunogold labeling confirmed the human renin localization within restored dense core granules.

Data from the present study also supports the hypothesis that the level of dense core granulation formation is dependent on the expression level of renin protein. In the *hRen*^+/−^*Ren1d*^−/−^ animals, a slight nonsignificant gender-based increase in basal *mRen2* expression was seen in the females. However, females showed significantly increased *hRen* transgene expression levels, giving us an effective system in which to investigate the process of granulation at different expression levels without confounding RAS interactions. This difference was also seen in a further, independent transgenic line (1317 *hRen*^+/−^*Ren1d*^−/−^).^5^

In male *hRen*^+/−^*Ren1d*^−/−^ animals, rescue of the granulation of the JG cells was blunted compared with females, which was mirrored in the *hRen* expression levels. 3D reconstructions of JG cells in untreated *hRen*^+/−^*Ren1d*^−/−^ mice revealed that females formed a larger number of granules, which occupied significantly more extranuclear volume than males and were more regular in shape. These data suggest that the basal level of *hRen* expression in JG cells of male mice is insufficient to drive complete granulation, but that up-regulation of human renin expression allows increased granulopoiesis. A number of electron-lucent granules from male *hRen*^+/−^*Ren1d*^−/−^ mice were visualized in 2D and 3D and shown to contain areas of paracrystalline material, particularly after captopril treatment. This suggests incomplete granule formation and may show granules in the process of coalescing (1).

*hRen*^+/−^*Ren1d*^−/−^ mice responded appropriately to pharmacological manipulation of the RAS via chronic ACE inhibition, as shown by the increase in human renin expression levels. The relative insensitivity of *mRen2* expression to the administration of captopril is likely to be due to the fact that expression and secretion of *mRen2* is not controlled by the regulated release pathway of active renin, because it is constitutively released. This is supported by the observation that, in humans, acute administration of ACE inhibitors causes either a fall in prorenin secretion, or no change at all, whereas active renin is significantly elevated (16, 17).

Treatment with captopril induced increased renin expression and granulation within JG cells. Treated *Ren1d*^−/−^ mice showed dilated RER as well as the appearance of electron-lucent granules and vacuoles in JG cells. This is particularly interesting because electron-lucent granules were observed even though no active renin was able to be synthesized. This may suggest that when the RAS is strongly stimulated, granule formation is initiated irrespective of renin transcription, perhaps to facilitate demand for renin when perfusion pressure drops (18).

Similar features were also present in captopril-treated *hRen*^+/−^*Ren1d*^−/−^ mice, where the increased number of granules exhibited a wider range of electron-lucency compared with untreated *hRen*^+/−^*Ren1d*^−/−^ JG cells. This diversity of granule electron densities has also been documented in C57Bl/6 mice after perfusion with isoproterenol and EGTA to stimulate secretion (2, 19). Granule electron densities were categorized into normal, vesicles in transition from normal to lower density, low density, and emptied, all of which are visible in captopril-treated *hRen*^+/−^*Ren1d*^−/−^ JG cells. The appearance of electron-lucent granules were attributed to emptying of existing granules rather than production of new granules, due to their location at the cell periphery and the short incubation time used in the study, insufficient for the production of new granules (2, 3). Furthermore, short-term administration of the ACE inhibitor enalapril was reported to induce rapid secretion of renin from JG cells, degranulating mature granules (19).

The alterations in granule morphology seen after captopril treatment in JG cells from *hRen*^+/−^*Ren1d*^−/−^ and *Ren1d*^−/−^ mice are also consistent with those documented in C57Bl/6 animals after enalapril treatment for 7 days (19, 20). JG cells from enalapril-treated mice showed an increased number and range of granule morphologies from small and round to large, irregular shaped granules. Well developed Golgi cisternae had protogranules associated with them, dilated RER were observed and fusing granules were present, all of which were also observed in *hRen*^+/−^*Ren1d*^−/−^ mice. After 14 days treatment, a further increase in granule number and granular fusion was observed, as was further development of the Golgi cisternae and the RER. Similar vacuoles containing membrane-like material akin to those seen in *Ren1d*^−/−^ JG cells were also present, and vacuoles were seen in JG cells at all stages of enalapril treatment, although at no point was fusion of granules with the membrane observed. This would suggest that the electron-lucent granules seen in our chronically captopril-treated *hRen*^+/−^*Ren1d*^−/−^ mice were a combination of emptied renin granules and *de novo* granule formation, suggested by the presence of coalescing paracrystalline dense core material within granules.

The data presented would suggest that the extent of granulation within JG cells is dependent on the amount of renin transcribed; in *Ren1d*^−/−^ JG cells where no glycosylated protein is present no granules are generated, whereas the low transcription levels of renin in male *hRen*^+/−^*Ren1d*^−/−^ mice generated significantly less granulation than in the females with higher transcription levels. However, when the system is stressed, as it is under the chronic administration of captopril, intracellular structure becomes disorganized and electron lucent granules become much more abundant, both in *hRen*^+/−^*Ren1d*^−/−^ mice where renin transcription is high, and in *Ren1d*^−/−^ mice where no renin protein is being trafficked into dense core granules. This would suggest that the granules and the lysosomal enzymes within are up-regulated to such a degree that granules begin to be formed.

Complementation of the *Ren1d*^−/−^ mouse with human renin does not rescue atypical MD morphology phenotype, suggesting this phenotype is related to a feature of renin-1d enzyme activity, either as part of a paracrine RAS in the juxtaglomerular region of the kidney or through it's role in the systemic RAS.

The close anatomical relationship between the MD and JG cells of the afferent arteriole allows the autoregulation of glomerular circulation in response to the composition of fluid passing the MD plaque (21). Low sodium concentration at the MD stimulates renin release through the transcription and secretion of the signaling molecule prostaglandin E2, which stimulates cAMP-mediated renin transcription and secretion from JG cells (22–24). Conversely, high tubular sodium concentration at the MD initiates an ATP-dependent Ca^2+^ wave that travels via gap junctions throughout the JGA and beyond into the intraglomerular space (15). This participates in TGF by contracting the glomerular tuft and the afferent arteriole to reduce GFR (15).

*Ren1d*^−/−^ animals display abnormalities in the MD cell morphology, irrespective of human renin complementation, with an alteration in cell number and shape compared with control animals. It is likely that the abnormal MD structure is a result of perturbations in the RAS, possibly as a response to maintain appropriate blood pressure in these animals. This suggestion is supported by the slightly hypotensive phenotype observed in female *Ren1d* knock-out animals (11) and the lack of rescue of the abnormal MD morphology by complementation of *Ren1d*^−/−^ animals with the human renin protein, which is unable to cleave mouse angiotensinogen (25) to initiate the RAS cascade. However, at present the sequence of events leading to the abnormal MD morphology phenotype is not understood.

In conclusion, we have shown that the human renin protein is able to complement the mouse *Ren1d*^−/−^ non-granulated defect and suggests that granulopoiesis requires a structural marker that is conserved between the mouse and human renin proteins. However, it also suggests that MD phenotype is dependent on the activity of the renin-1d enzyme in a local juxtaglomerular renin-angiotensin system.

## Experimental procedures

Experiments were approved by the University of Edinburgh Animal Welfare and Ethical Review body (AWERB) and were conducted in accordance with the Animals (Scientific Procedures) Act 1986 and the Guiding Principles for Research Involving Animal and the Institutional Animal Care and Use Committee of the University of Southern California.

### Generation of hRen-complemented Ren1d^−/−^ mice

PAC 111L11 was obtained from Genome Systems, Inc. This clone contained the human *Ren* gene on chromosome I with ∼35 kb of flanking sequences upstream and 90 kb of flanking sequences downstream of the structural genes (25). PAC DNA was digested with NotI (Roche Applied Science) and a 55-kb fragment spanning the human renin locus (transgene *hRen*) purified by preparative pulse-field gel electrophoresis, β-agarose treatment (New England Biolabs), and dialysis against injection buffer (10 mm Tris-HCl, pH 7.5, 0.1 mm EDTA, 100 mm NaCl). DNA at 1 mg/ml was microinjected using standard techniques into fertilized C3H × CBA/Ca F1 oocytes (*Ren1c* background). Transgene-positive animals were identified by PCR and BamHI digestion of tail DNA, which was analyzed by Southern blot hybridization using a *Ren2* cDNA probe.

A breeding strategy was designed to introduce the human renin transgene onto the *Ren1d*^−/−^ background (Fig. 1*B*). A transgenic animal hemizygous for the *hRen* transgene (on a *Ren1c* background) was crossed to a *Ren1d*^−/−^ mouse. The progeny were obligate heterozygotes for *Ren1c* and *Ren1d*. F1 animals hemizygous for the *hRen* transgene were backcrossed to homozygous *Ren1d*^−/−^ animals.

### Transgenic animal ACE inhibition studies

Both male and female mice from lines *hRen*^+/−^*Ren1d*^−/−^ and *Ren1d*^−/−^ (*n* = 6) were treated for 10 days with captopril (Sigma), administered at a concentration of 1 mg/ml in drinking water.

### Transmission electron microscopy ultrastructure analysis

Kidneys were perfusion fixed at a constant pressure (120 mmHg, 5 min) with 2.5% glutaraldehyde in 0.1 m phosphate buffer (pH 7.2). Tissue was stored in this fixative for 4 h at room temperature before being transferred to a 10-fold dilution of buffer and stored at 4 °C before preparation for electron microscopy by standard methods (26). Briefly, cells were post-fixed in osmium tetroxide (1% (w/v) in 0.1 m phosphate buffer), then stained with uranyl acetate (2% (w/v) in distilled water), dehydrated through increasing concentrations of ethanol (70–100%), and embedded in Spurr resin (Agar Scientific). Semi-thin sections were cut and stained in toluidine blue for specimen orientation. Ultrathin sections (50–80 nm) were prepared using a Reichert Ultracut S Microtome, mounted on 200-mesh nickel grids, and stained lightly with uranyl acetate and lead citrate. Grids were viewed on a JEOL transmission electron microscope (JEM-1010, JEOL, Peabody, MA).

### Immunogold labeling

Kidneys were perfused with heparinized saline for 3 min, followed by tannic acid (0.2% (w/v) tannic acid in PBS, 10 min), then perfusion fixed with freshly prepared 3% paraformaldehyde in 0.1 m phosphate buffer (pH 7.2) for 5 min. Kidneys were removed and stored at room temperature in this buffer for 4 h before being transferred to a 10-fold dilution and stored at 4 °C. Sections were prepared for immunogold electron microscopy by standard methods (27). Briefly, segments were stained with uranyl acetate (2% (w/v) in distilled water), dehydrated through increasing concentrations of methanol (70–100%), and embedded in LR Gold (London Resin Company). Ultrathin sections (50–80 nm) were prepared as above, incubated at room temperature for 2 h with anti-human renin (Human renin affinity purified polyclonal Ab, R&D Systems, AF4090, 1:1000) or anti-mouse renin (raised against mouse Ren 2 in rabbit, Inagami (28), 1:10,000), and for 1 h with Protein A–15 nm gold complex (British Biocell). All antisera were diluted in 0.1 m phosphate buffer containing 0.1% egg albumin. As a control the primary antibody was replaced by phosphate buffer/egg albumin. After immunolabeling, sections were lightly counterstained with lead citrate and uranyl acetate, and were imaged with a JEOL transmission electron microscope as above.

### 3D reconstruction of JG cells

Kidneys were perfusion fixed at a constant pressure (120 mmHg, 5 min) using 2% gluteraldehyde (Sigma) in 0.1 m cacodylate (Agar Scientific) buffer. Embedding for TEM was then performed at the University of Regensburg as previously described (2). Briefly, an automatic microwave (Leica EM, Germany) was used to embed tissue segments in epoxide resin, which were then sectioned serially to 70-nm ultrathin sections using an ultramicrotome (EM UC7, Leica, Germany). They were then transferred to copper grids coated with pioloform and contrast-stained using 4% uranyl acetate and lead citrate solution. Images were acquired using a Phillips CM12 TEM with a LaB_5_ cathode and an acceleration voltage of 120 keV. Digitalization was performed using a TEM-1000 slow-scan CCD camera and EM-Menu 4.0 software.

Images were stacked sequentially in ImageJ and loaded into Amira 3D Software for Lifesciences (FEI). JG cells were reconstructed according to a method described by Steppan *et al.* (2). Briefly, slices were aligned for each cell of interest, scaled, resampled, and the nucleus, membrane, and granules manually segmented. Surfaces were then rendered from the label fields and assigned to different materials. The volume of each of these materials was computed and a 3D model of the JG cell produced.

### H & E staining and imaging

Kidney tissue (*n* = 4–5) was decapsulated and immersion-fixed in 4% paraformaldehyde and subsequently embedded in paraffin wax blocks. 5-μm sections were taken and stained with hematoxylin and eosin and blindly examined using an Olympus BX51 upright microscope with a ×40 air UPlanSApo 0.95 NA lens at room temperature. Images were captured using a QImaging Micropublisher 3.3 RTV camera and QCapture Pro7, and analyzed using Fiji.

### Plasma renin activity levels

Blood was collected by cardiac puncture immediately after cervical dislocation. Mouse and human activity radioimmunoassays were performed separately; mouse renin activity was determined as previously described (29), and human renin activity was determined using the Adaltis Renin radioimmunoassay kit (Ref. 12964, Adaltis, Italy).

### RNA extraction and quantitative real-time PCR analysis

Total RNA was isolated from frozen tissue using an RNeasy Mini Kit (Qiagen) according to the manufacturer's instructions. Kidney was homogenized in RLT buffer by shaking with a metallic homogenization bead for 2 min at 30 Hz in a Retsch MM301 tissue disrupter (Haan). Genomic DNA was removed using a DNA Free Kit (Ambion) according to the manufacturer's instructions, and the RNA integrity verified using a Nanodrop and gel electrophoresis. cDNA synthesis was carried out using a High Capacity cDNA Reverse Transcription kit (Applied Biosciences) according to the manufacturer's instructions.

Human and mouse renin gene transcription was assessed by quantitative real-time PCR in a volume of 10 μl and monitored on a Roche Lightcycler 480 System using the Universal Probe Library. The primers and probe used for the assays were designed using the Roche Universal Probe Library Assay Design Centre and were obtained from Eurofins Genomic (EU). mRNA levels were normalized to tbp and 18S. Human renin (UPL probe 77) forward primer, 5′-tacctttggtctcccgacag-3′, reverse primer, 5′-ttgagggcattctcttgagg-3′; mouse renin (UPL probe 16) forward primer, 5′-cccgacatttcctttgacc-3′, reverse primer, 5′-tgtgcacagcttgtctctcc-3′; tbp (UPL probe 97) forward primer, 5′-gggagaatcatggaccagaa-3′, reverse primer, 5′-gatgggaattccaggagtca-3′; 18S (UPL probe 77) forward primer, 5′-ctcaacacgggaaacctcac-3′, reverse primer, 5′-cgctccaccaactaagaacg-3′.

### In vitro isolated and microperfused AA-JGA-glomerulus

Male C57Bl/6, male *Ren1d*^−/−^, and female *hRen*^+/−^*Ren1d*^−/−^ adult mice were anesthetized with 10 mg/100 g of body weight with ketamine/xylazine (1:1) and the left ventricle perfused with ice-cold PBS. Kidneys were removed, decapsulated, and glomeruli with attached AA and DT containing the MD microdissected (13). Briefly, dissection media comprising DMEM/F-12, 1.2 g/liter NaHCO_3_, 3% FBS (pH 7.4) was prepared and aerated with 95% O_2_, 5% CO_2_ for 45 min prior to perfusion. Arteriole perfusate comprised a modified Krebs-Ringer-HCO_3_ buffer (mm: 115 NaCl, 5 KCl, 25 NaHCO_3_, 0.96 NaH_2_PO_4_, 0.24 Na_2_HPO_4_, 1.2 MgSO_4_, 2 CaCl_2_, 5.5 d-glucose, 0.1 l-arginine), maintained at ∼50 mm Hg (1 p.s.i.). This buffer was also used as bathing solution, exchanged at a rate of 1 ml/min. Tubular perfusate comprised an isosmotic, low-NaCl, Ringer solution (mm: 10 NaCl, 135 *N*-methyl-d-glucamine-cyclamate, 5 KCl, 1 MgSO_4_, 1.6 Na_2_HPO_4_, 0.4 NaH_2_PO_4_, 1.5 CaCl_2_, 5 d-glucose, 10 HEPES), perfused at a baseline rate of 2 nl/min. Solutions were kept at 4 °C throughout cannulation of the arteriole and tubule, then raised to 37 °C. TGF activation was performed via increased tubular perfusion from 2 to 20 nl/min using a constant 10 mm [NaCl] perfusate (flow-induced TGF). Tubular perfusate was kept isosmotic by reducing *N*-methyl-d-glucamine-cyclamate to 65 mm.

### Confocal laser scanning fluorescence microscopy

Imaging of calcium transits was performed using a Leica TSC SP2 AOBS MP confocal microscope system (Leica, Heigelberg) in a *xyt* time series at 1 frame/2 s, using a 488-nm 20-milliwatt laser to power a Leica DM IRE2-inverted microscope and Leica LCS imaging software. Calibration of tubular perfusate was previously performed by the Peti-Peterdi laboratory (13), allowing absolute calcium values to be obtained from fluorescence intensity (12 bit) measurements using Fluo-4 AM (excitation/emission: 488/520) and Fura Red AM (excitation/emission: 488/650). Gain, offset, and laser power were kept constant between experiments.

Preparations were loaded with both dyes (10 μm, Invitrogen), by dissolving them in DMSO and adding them to both the tubular and arteriolar perfusate and loading them for ∼15 min. After 20 min of fluorescent signal stabilization. Fluo-4 to Fura Red ratios (R) were converted to absolute [Ca^2+^]*_i_* values with an intracellular calibration method as described previously (13, 30, 31, 32).

### Statistics

Data were analyzed by Student's *t* test with Tukey's post hoc analysis and the level of significance was set to *p* < 0.05. Error bars represent S.E. with *, *p* < 0.05; **, *p* < 0.01; ***, *p* < 0.001; ****, *p* < 0.0001 by two-way analysis of variance in conjunction with Bonferroni post hoc analysis.

## Author contributions

C. B., L. J. M., M. G. S., S. F., and S. S. resources; C. B., D. S., and A. K. software; C. B., D. S., J. P.-P., and H. C. formal analysis; C. B. validation; C. B., R. J. N., L. J. M., S. F., C. J. K., J. P.-P., and H. C. investigation; C. B., S. F., D. S., J. P.-P., and H. C. visualization; C. B., R. J. N., L. J. M., M. G. S., S. S., J. P.-P., H. C., and J. J. M. methodology; C. B., R. J. N., S. S., H. C., and J. J. M. writing-original draft; C. B. and J. J. M. project administration; C. B., L. J. M., M. G .S., S. F., C. J. K., S. S., D. S., J. P.-P., A. K., H. C., and J. J. M. writing-review and editing; R. J. N. and J. J. M. conceptualization; R. J. N., S. F., S. S., and H. C. data curation; L. J. M., M. G. S., J. P.-P., A. K., and J. J. M. supervision; J. J. M. funding acquisition.

## Footnotes

5 R. J. Nelson and C. Buckley, unpublished observations.

## Footnotes

4 The abbreviations used are:

Ang: angiotensin
ACE: angiotensin-converting enzyme
JG: juxtaglomerular
MD: macular densa
TGF: tubuloglomerular feedback
JGA: juxtaglomerular apparatus
TEM: transmission electron microscopy
AA: afferent arteriole.
